# Population Dynamics of Off-Host *Rhipicephalus (Boophilus) microplus* (Acari: Ixodidae) Larvae in Response to Habitat and Seasonality in South Texas

**DOI:** 10.3390/vetsci5020033

**Published:** 2018-03-23

**Authors:** Brenda Leal, Donald B. Thomas, Robert K. Dearth

**Affiliations:** 1 U.S. Department of Agriculture, Agricultural Research Service, Cattle Fever Tick Research Laboratory, Edinburg, TX 78541, USA; donald.thomas@ars.usda.gov; 2 Department of Biology, University of Texas Rio-Grande Valley, Edinburg, TX 78539, USA; robert.dearth@utrgv.edu

**Keywords:** cattle tick, ecology, habitat, climatic factors

## Abstract

The cattle tick, *Rhipicephalus microplus* (Canestrini), is an economically destructive arthropod because of its ability to vector bovine babesiosis. It is known that cattle ticks can spend 80–90% of their lifecycle as questing larvae, yet the effect of climatic factors on their off-host behavior and survival is unclear. The goal of this study was to measure the effects of specific ecological factors on off-host questing larvae in nature. The study was conducted in a south Texas pasture over a two-year period, during which time larval populations were surveyed. Simultaneously, weather variables—precipitation, relative humidity, and ambient temperatures—were recorded. Larval survival rates varied among seasons, with the overall highest populations recorded in the spring and the lowest in the fall by a ratio of 20:1. In the winter, the larger numbers were collected from exposed habitats at a ratio of 6:1. Conversely, canopied habitats in the summer had 10-fold larger larval numbers. In the spring, exposed and canopied habitats showed no difference in tick larval survival rates. The results show that the interaction between season and habitat strongly influence off-host questing tick survival. Relative humidity was a key weather variable.

## 1. Introduction

The cattle fever tick, *Rhipicephalus (Boophilus) microplus* (Canestrini), is a one-host tick known to transmit hemoparasites that cause bovine babesiosis and anaplasmosis [1,2,3,4]. In the early 1900s, babesiosis devastated the cattle industry and was detrimental to the U.S. economy. This led to the eradication of the cattle fever tick in the USA by 1943 [5]. Unfortunately, infestations periodically recur along the south Texas border threatening the region and serving as a gateway to spreading the disease northward [6]. Currently, acaricides are applied directly to cattle to prevent and destroy ticks [7]. However, poorly monitored and free-roaming hosts have been identified (nilgai, white-tail deer) [8]. This, coupled with the knowledge that the majority of the *Boophilus* life-cycle is off-host, complicates current control practices. Thus, efforts to better understand the *B. microplus* life cycle and create broader management practices are vital.

The life cycle of *Boophilus* ticks has seven stages: pre-oviposition adult, ovipositing adult, incubating eggs, questing larva, attached larva, nymph, and feeding adult [7]. One-host ticks will spend part of their larval stage off-host, then once attached will feed and develop on just one host. Typically, as much as 80% of the life cycle consists of the larvae questing for a host. Questing is tick behavior that consists of climbing from the ground up into the vegetation, clustering on grasses (or other pasture plants), extension of the front legs where the sensory organs are located, and waiting for a potential passing host [8,9]. Questing is costly; ticks lose energy and water, and receive no nourishment. Yet, questing becomes the key to tick survival. Without a host, the tick dies. Surprisingly, in some cases, Ixodid larvae can survive off-host 8–9 months even in harsh semi-arid environments with limited water and high temperatures before dying from starvation [10,11,12,13]. Therefore, a better understanding of the ecological conditions that influence questing behavior and tick survival could be used to predict outbreaks and develop off-host prophylactic control strategies.

Questing behavior and larval survival has been shown to be influenced by multiple factors [14,15]. Previous studies have shown that seasonality, ambient and soil temperatures, humidity, sun exposure, vegetation and precipitation effect survival. Overall, temperature and humidity have the greatest influence on questing behavior of tick larvae [16]. These climatic factors fluctuate within and among seasons, influencing the length (increase or decrease) of the questing period [17]. Furthermore, habitat topology has been shown to affect the severity of the impact that temperature and/or humidity has on larval survivability and questing behavior.

Our current knowledge of the climatic factors that affect cattle tick survival is largely based on extensive ecological studies conducted in Australia on what was believed to be *Rhipicephalus* (*Boophilus*) *microplus*. But recent cross-mating and genetic studies have resulted in the separation of *Rhipicephalus australis*, the species studied in Australia [18,19,20] (*Boophilus* is now considered to be a subgenus of *Rhipicephalus*). Consequently, there is a paucity of information based on field studies of *R. microplus*, the species in North America. The Australian climate is different from the south Texas climate, signifying a need for more ecological studies on the *R. microplus* complex, including the predominant fever tick species in south Texas.

An ecological model was developed by Teel et al. [8] for south Texas climate regimes [21], using field data from Australia and lab studies on fever tick response to environmental variables. The model’s intent was to predict the influence of season and habitat on Texas populations [8]. However, it is unclear how well these predictions translate to reality given the confounding taxonomic problems. Therefore, in our current study, we observed the interaction between seasonality, habitat, and questing populations of *R. microplus* in natural conditions. The goal of this study was to validate the predicted effects of specific ecological factors on off-host questing larval survival in south Texas. Furthermore, this study will strengthen our understanding of *R. microplus* larvae and create a foundation for future ecological studies.

## 2. Materials and Methods

### 2.1. Study Site

This study was conducted at the United States Department of Agriculture (USDA)-Agricultural Research Service, Cattle Fever Tick Research Laboratory in a pasture at Moore Air Field located near Edinburg, TX, USA (26.3871° N, 98.3376° W; elevation 66 m). The Lower Rio Grande Valley is a semi-arid and subtropical region with ambient temperatures averaging between lows of 8 °C in the winter to highs of 36 °C in the summer [22]. The valley, which is more of a fertile plain than a valley, has an annual rainfall of 38–75 cm that is highly erratic both seasonally and annually [23,24,25]. The experimental pasture contains vegetation characterized as Tamaulipan brushland [25]. The soil is a shallow calcareous clay with caliche near the surface. Vegetative cover overall is around 90%, with a canopy cover of around 20%. The dominant tree species is honey mesquite, *Prosopis glandulosa* (Torr.), with shrubby acacias, *Vachellia rigidula* (Benth.), *Vachellia farnesiana*, and spiny hackberry, *Celtis ehrenbergiana* (Klotzsch). Typical of pastureland of south Texas, the dominant understory plant is buffelgrass, *Pennisetum ciliare* (L.), with the common forbs cowpen daisy, *Verbesiana encelioides* (Cav.) and silverleaf nightshade, *Solanum elaeginifolium* (Cav.), mainly in open, disturbed areas. Plant names follow the USDA Plant Database. [26].

### 2.2. Rearing of Ticks

Ticks were reared as described previously [27]. Briefly, larval ticks were placed on stanchioned cattle at the USDA quarantine facility and allowed to develop until females were engorged and dropped from the host. These females were held in petri dishes (at 27 ± 1 °C, 80 ± 5 Relative Humidity (RH)) for oviposition. Experimental colonies of *R. microplus* were maintained under optimal conditions in a climate-controlled room [7,28]. The strain designated as “Deutch” in generations F59, F60 and F61 was used to infest gardens as described below.

### 2.3. Tick Gardens

**Figure 1:**
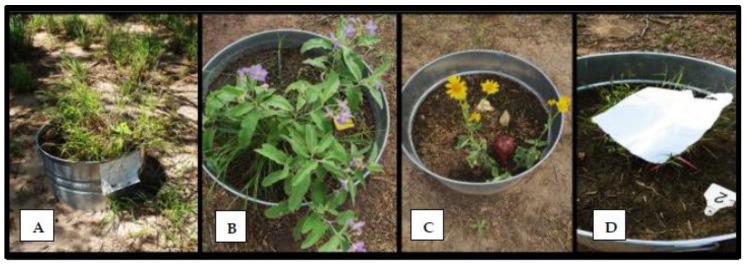
Tick gardens were infested with one female at the start of each cohort: (**A**) buffelgrass; (**B**) silverleaf nightshade; (**C**) cow-pen daisy; (**D**) white flannel sheet (25 × 20 cm) used to collect larvae.

**Figure 2:**
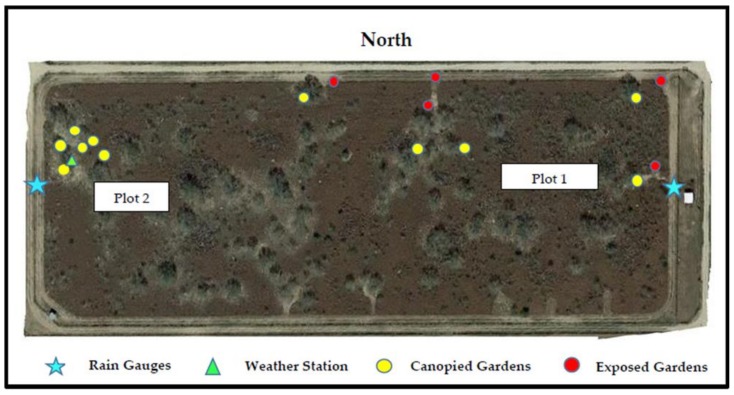
Satellite photo of eight-hectare study pasture showing the juxtaposition of canopied and exposed tick gardens. Plot 1 is located on the north and plot 2 is located on the west side. In plot 1, all tick gardens contained bufflegrass and plot 2 had 2 bufflegrass, 2 nightshades and 2 daisies. The weather station was located in plot 2.

Study arenas “tick gardens” consisted of 18 individual metal tubs (American Metalcraft, Franklin Park, IL, USA) filled three-quarters to the rim with soil. Each was planted with one of three common south Texas pasture plants: buffelgrass (Figure 1A), silverleaf nightshade (Figure 1B), and cowpen daisy (Figure 1C). These plants were ideal for these conditions because they thrive in areas with low precipitation. Buffelgrass, *Pennisetum ciliare* (L.) is an invasive dominant pasture grass in south Texas and northern Mexico, native to Africa [29]. Silverleaf nightshade, *Solanum elaeagnifolium*, is a native plant to south Texas that contains spines with a sticky texture [30]. Cowpen daisy or yellow-top, *Verbesina encelioides*, is also native to south Texas. It grows throughout the year as long as winter conditions are mild [31]. A total of 16–18 tick gardens were scattered throughout the eight-hectare pasture. The gardens were divided into two plots (Figure 2). The first plot, on the north and east side of the pasture, consisted of 10 gardens divided equally into five exposed and five canopied habitats. The second plot, on the west end of the pasture, consisted of six gardens all placed in a canopied habitat. The gardens in the first plot were all planted with buffelgrass, whereas the gardens in the second plot were a mix of three plant species: silverleaf nightshade (2–4), cowpen daisy (2–4), and buffelgrass (3).

### 2.4. Data Collection

At the beginning of every replicate, each tick garden was infested with one engorged female from the colony, then placed in the center of the tub by the stem of the plant. Once placed, these females were not disturbed and allowed to complete oviposition over a three- to four-week period. Data were collected using the standard flag method [32]. A white flannel cloth (dimensions 25 × 20 cm) was placed directly over the plant then dragged in opposite directions to represent a potential passing host (collection time approximately 40 s) (Figure 1D). Each flannel cloth was then placed in a numbered zip-lock bag corresponding to each tub. Following the recording methods of Wilkinson [32], larvae attached to the cloth were collected with clear adhesive tape then mounted directly on a data sheet. Twelve censuses were taken per month, with one to three days between each census. All gardens were sampled at each collection date. Sampling clock-times were varied to include all periods of day and night. Data were collected continuously over a two-year period. Abiotic factors were measured by two rain gauges, one on each end of the pasture to measure precipitation levels, and a HOBO Pro model V2 micro weather station (Onset Computer Corporation, Bourne, MA, USA) to record ambient temperatures.

### 2.5. Cohorts

**Figure 3:**
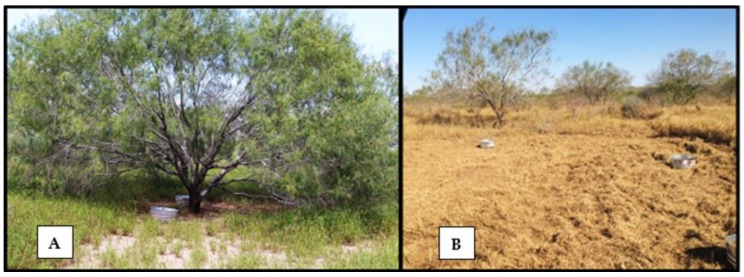
(**A**) Canopied habitats consisted of large trees used to provide complete coverage to gardens; (**B**) Exposed habitats contained gardens directly exposed to sunlight.

At regular intervals during the study, a full set of tick gardens were infested and the populations monitored. Each set of tick gardens were designated as a cohort. If a plant died, it was replaced between cohorts. Cohorts were separated into four seasonal categories: winter (November–February), summer (June–August), spring (March–May), and fall (September–October). Within each cohort, tick gardens were placed in either canopied or exposed habitats (Figure 3A,B). For each garden in each cohort we measured the time interval from the introduction of females to the first positive larval sample, to the peak in the population, and lastly, date from first to the last positive larval sample. A new cohort would begin as the previous one ended. The cohorts ranged in duration from 34 to 109 days. In total (over two years), there were 13 cohorts: four cohorts in the winter, three cohorts in the summer, three cohorts in the spring, and three cohorts in the fall. A cohort was assigned to a season corresponding to the time of year the larvae appeared.

### 2.6. Statistical Analysis

Parameters measured for each cohort were as follows: total numbers of larvae per individual garden, total larvae per each positive garden, mean larvae per garden by cohort and for canopied and exposed habitats respectively, mean numbers of larvae per cohort by season, and percentage of gardens positive for larvae by cohort, by habitat, and by season. A “positive” garden was one in which larvae were detected, indicating survival and reproductive success by the released gravid female. Tests of the differences between mean numbers of larvae per garden by season and for canopied and exposed habitats were conducted by a Pair-wise t-test assuming unequal variance. Linear regression was used to measure correlation (*r*^2^) between larval numbers by cohort and the corresponding weather variables. For the Y-axis the weather variables were numerically expressed as mean-maximum (mean-max) or mean-minimum (mean-min) relative humidity (RH), mean-max and mean-min temperature, and total precipitation during each corresponding cohort. Additionally, each of the aforementioned weather variables were time constrained to either the egg incubation phase or the larval phase, of each corresponding cohort. The statistical significance of the correlation coefficient was calculated by ANOVA using the online program QuickCalcs (GraphPad Software, La Jolla, CA, USA) [33], which also generated the corresponding plots providing the slope and polarity (+/−) of the correlation. The data were tested for normality using the Shapiro-Wilk test [34].

## 3. Results

The data on mean number of larvae per garden approximated normality with the Shapiro-Wilk test (w = 0.82, α = 0.86). Variability was large among plant species such that there were no significant differences among the three plant species. Thus, data from the two plots were combined to increase sample size. The results show significant differences among seasons and between habitats (Table 1). Specifically, the fall cohorts were significantly lower in mean number of larvae per garden than those in winter, spring, and summer. This difference persisted when removing from consideration gardens that failed because females died without producing offspring (positive gardens only). In order of success in terms of population size, the seasons with the highest to lowest numbers of larvae were as follows: spring ($\bar{x}$ = 130.5), summer ($\bar{x}$ = 81.2), winter ($\bar{x}$ = 72.9) and fall ($\bar{x}$ = 6.6). In terms of female reproductive success (percentage of positive gardens) the order from highest to lowest was: spring (88%), winter (78%), summer (69%), and fall (54%). Interestingly, habitat had little effect on female reproductive success (Table 2). The overall percentage of positive gardens was not significantly different between canopied habitats and exposed habitats (*t* = 0.16, *p* = 0.86, df = 23) (Table 2). Thus it is not surprising that no correlation was found when comparing positive gardens to either RH or ambient temperature. There was no significant relationship between mean-max (*r*^2^ = 0.06, *p* = 0.39, *N* = 13) or mean-min (*r*^2^ = 0.02, *p* = 0.57, *N* = 13) temperatures or to mean-max (*r*^2^ = 0.02, *p* = 0.58, *N* = 13) or mean-min (*r*^2^ = 2.8 × 10^−5^, *p* = 0.39, *N* = 13) RH levels, nor to precipitation during the cohort (*r*^2^ = 0.01, *p* = 0.68, *N* = 13).

In contrast, habitat did have a strong effect on larval numbers within seasons. Numbers of larval ticks in the canopied habitat during fall ($\bar{x}$ = 7.3) and winter ($\bar{x}$ = 27.5) were significantly lower than they were in spring ($\bar{x}$ = 76.7) and summer ($\bar{x}$ = 111.6) (Table 1). However, canopied habitats in fall and winter were not significantly different (*t* = 1.81, *p* = 0.07, df = 50) from one another. Likewise, spring and summer were not significantly different from one another (*t* = 0.78, *p* = 0.43, df = 66). There were no differences in the mean larval numbers collected from exposed habitats in the fall and summer (*t* = 0.95, *p* = 0.34, df = 27). However, they were significantly (*p* < 0.05) lower when compared to winter or spring population numbers (Table 1). Winter and spring exposed habitat populations were not significantly different from one another (*t* = 1.12, *p* = 0.26, df = 71).

In winter, larval populations in canopied habitats were significantly lower than populations in exposed habitats (*t* = 2.52, *p* = 0.01, df = 23) by a factor of 6:1, and this was true each year. In the summer, populations in canopied habitats were significantly greater than populations in exposed habitats (*t* = 3.03, *p* ≤ 0.01, df = 33) by an order of magnitude. In the spring, larval numbers in canopied habitats were reduced but variable and not significantly different compared to numbers collected from exposed habitats (*t* = 1.63, *p* = 0.11, df = 26). Similarly, in the fall there was no significant difference in populations between canopied versus exposed habitats (*t* = 0.34, *p* = 0.73, df = 26).

**Figure 4:**
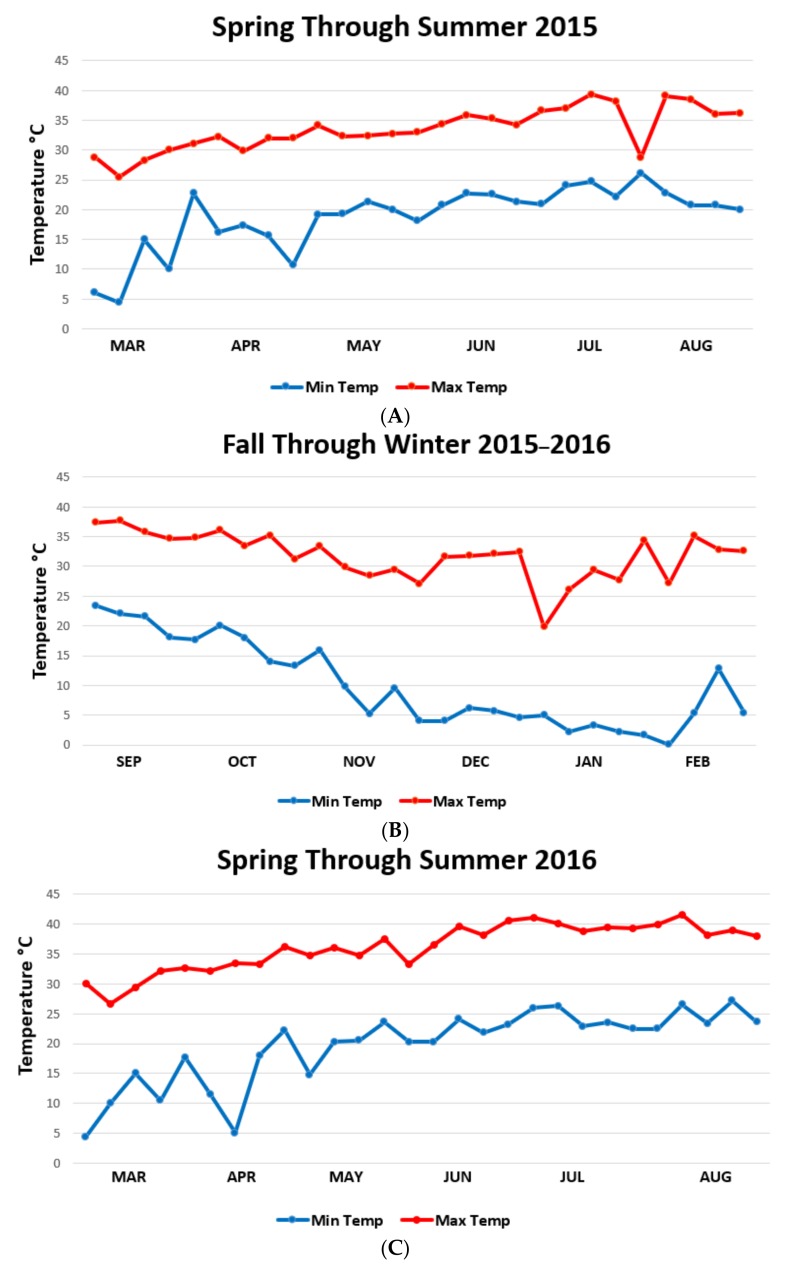

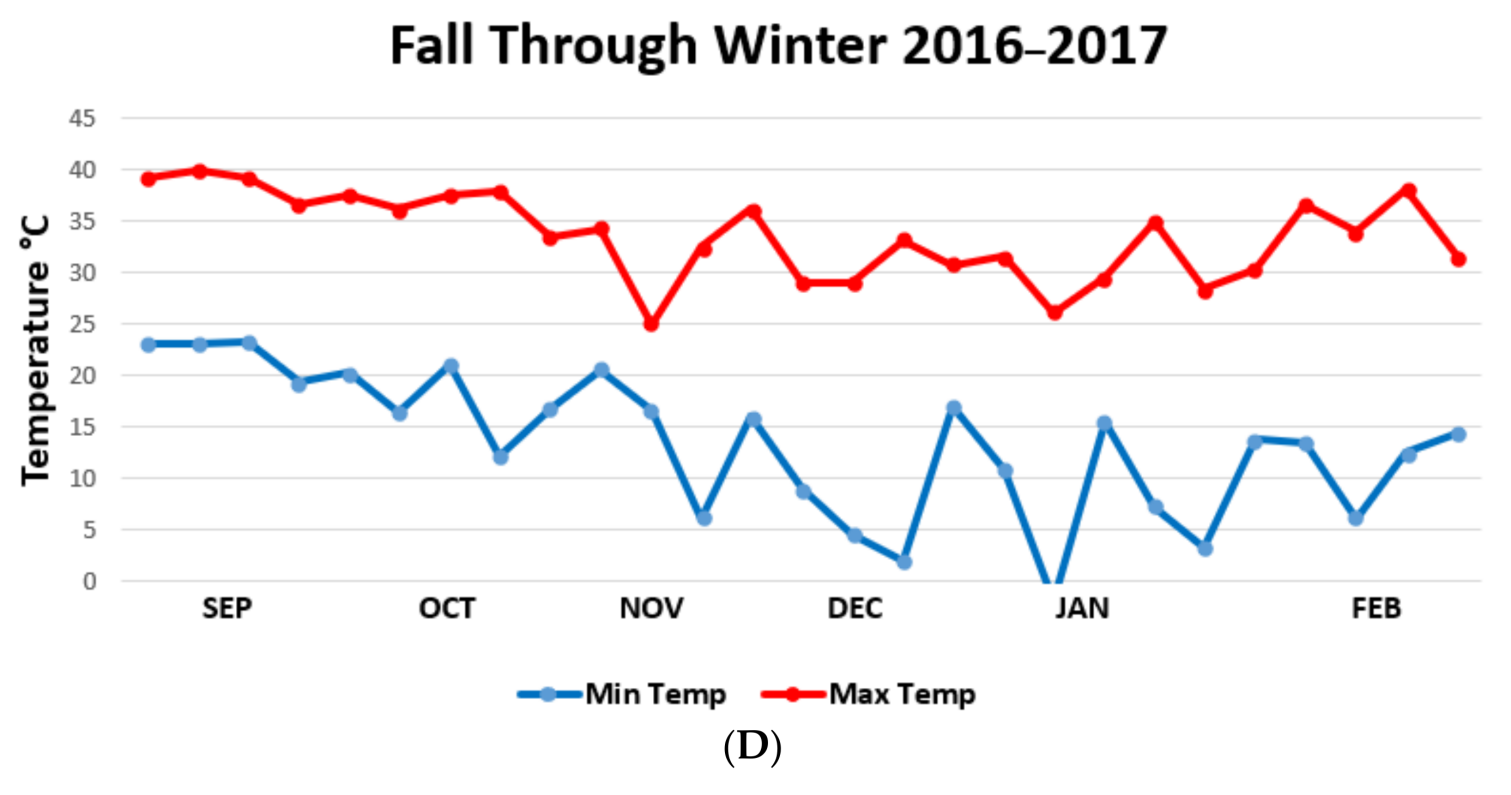
Mean-max and mean-min weekly ambient temperatures per every six months of sample collection. (**A**) March in the spring of 2015 to August 2015, next (**B**), September 2015 through February 2016, then (**C**), March in the spring of 2016 to August and finally (**D**) September in the fall of 2016 through February 2016.

**Figure 5:**
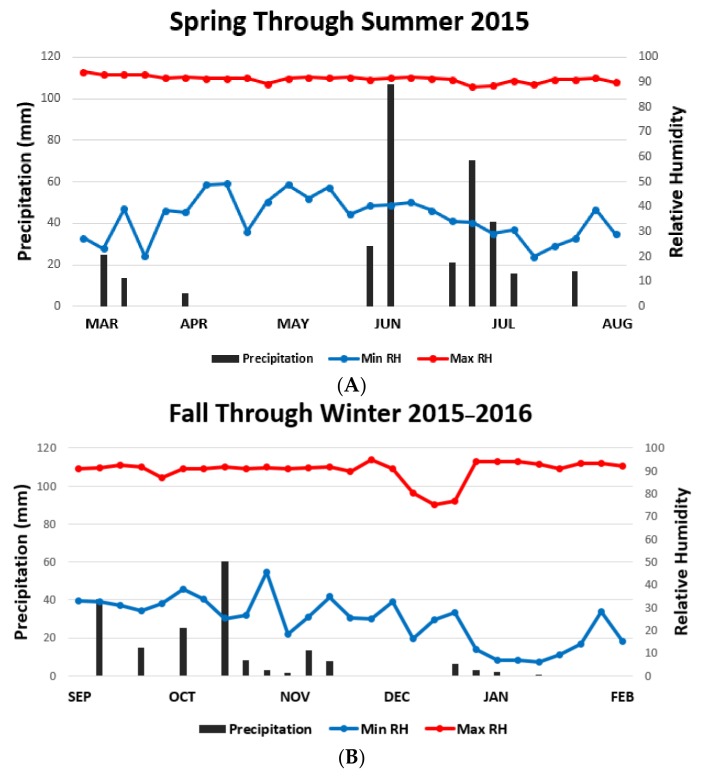

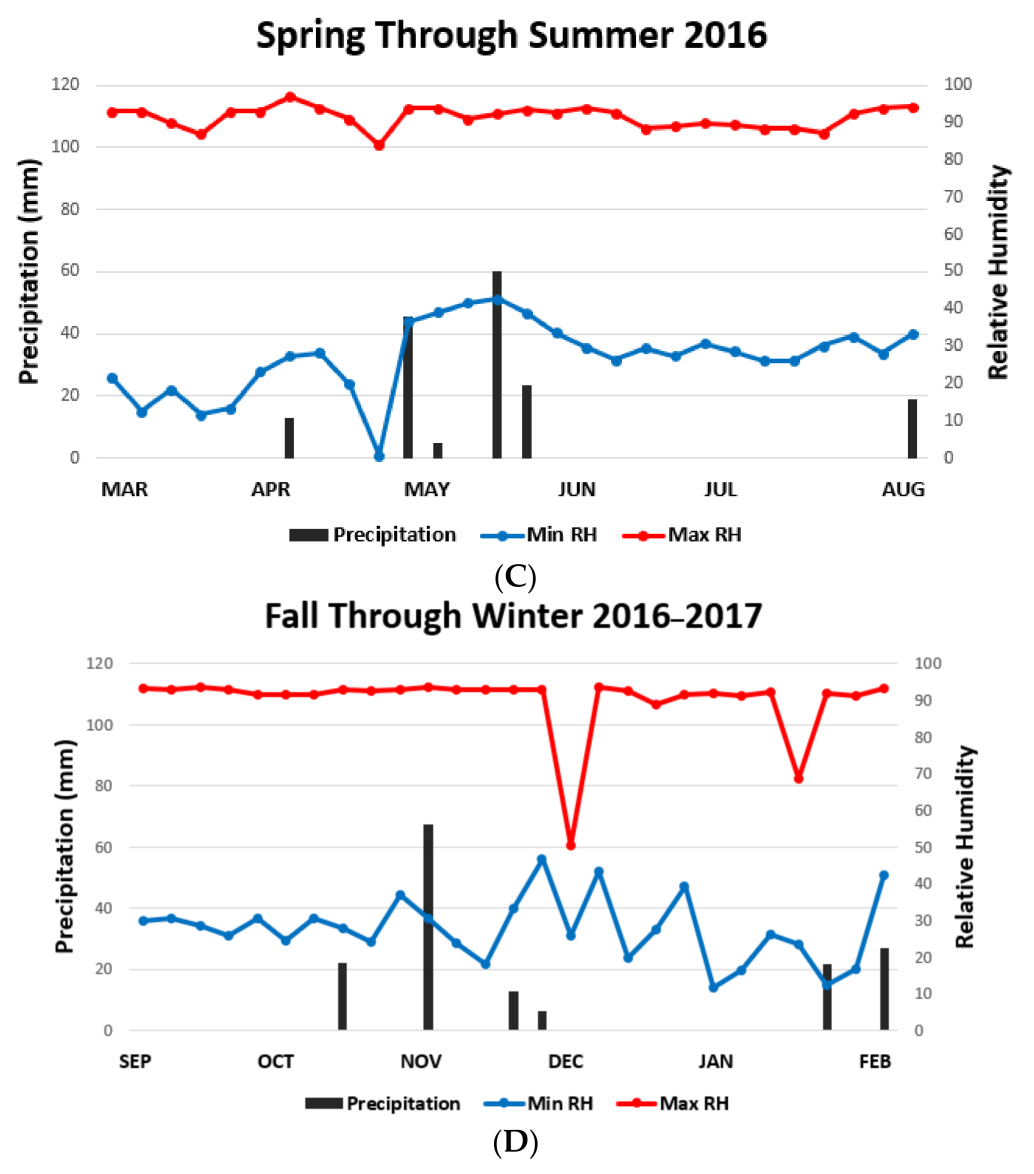
Precipitation events, mean-max and mean-min weekly relative humidity per every six months of sample collection (**A**) March in the spring of 2015 to August 2015, next (**B**), September 2015 through February 2016, then (**C**), March in the spring of 2016 to August and finally (**D**) September in the fall of 2016 through February 2016.

**Figure 6:**
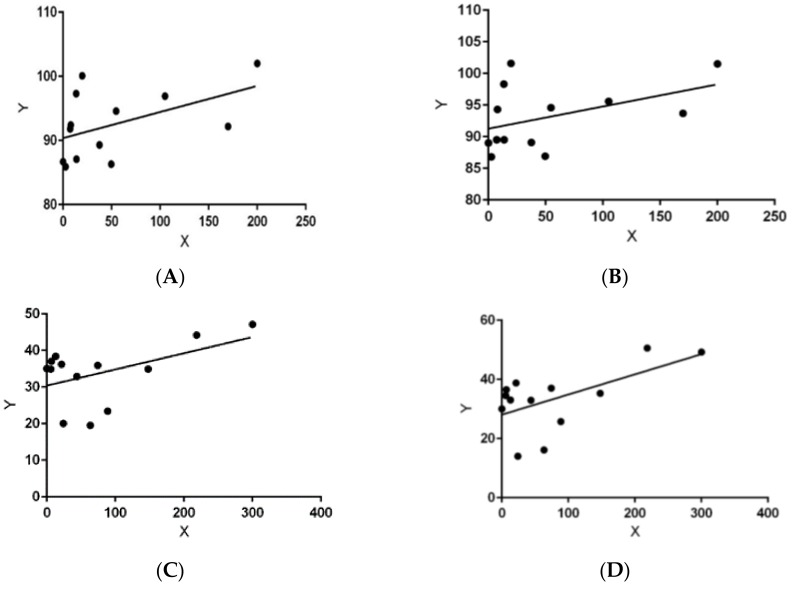

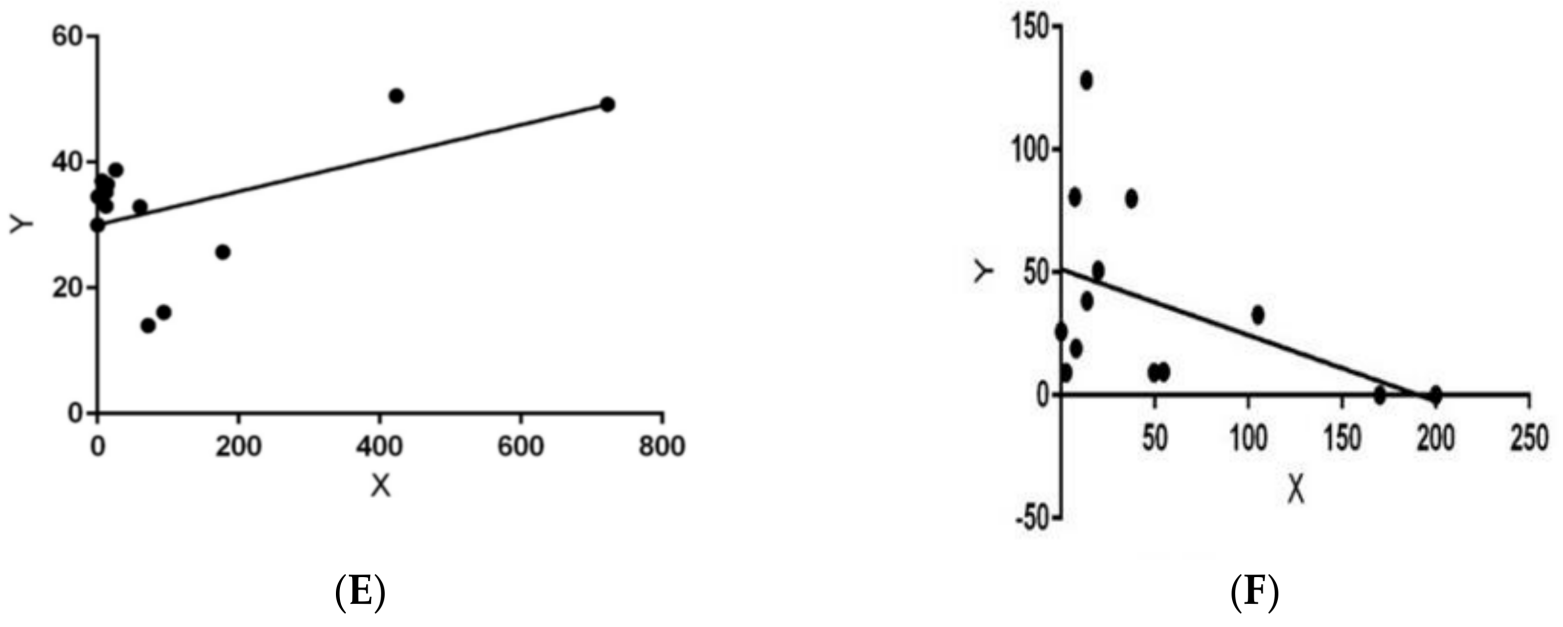
Regression graphs representing correlation between means of larvae, habitat, and abiotic factors. (**A**) Temperature in canopied gardens: X = ($\bar{X}$) larvae in canopied gardens, Y = mean-max temperature; (**B**) Temperature in canopied gardens during larval phase: X = ($\bar{X}$) larvae in canopied gardens, Y = mean-max temperature (larval phase); (**C**) Relative humidity (RH) in all habitats: X = ($\bar{X}$) larvae all habitats, Y = mean-min RH all cohort; (**D**) Relative humidity in all habitats during larval phase: X = ($\bar{X}$) larvae in all habitats, Y = mean-min RH (larval phase); (**E**) Relative humidity in exposed gardens during larval phase: X = ($\bar{X}$) larvae in exposed gardens, Y = mean-min RH (during larval phase); (**F**) Precipitation in canopied gardens during incubation phase: X = ($\bar{X}$) larvae in canopied gardens, Y = precipitation (incubation phase).

To explain these differences, we looked for correlations with weather patterns for all seasons within the two-year period (Figure 4 and Figure 5). Mean-max (*r^2^* = 0.05, *p* = 0.42, df = 1, 11) (Figure 6A) and mean-min (*r*^2^ = 0.01, *p* = 0.66, N = 13) ambient temperatures had no linear relationship to female reproductive success (percentage of positive gardens) under canopied habitats. However, there was a habitat-dependent effect of temperature on numbers. Temperatures during the larval phase had a marginal correlation with mean numbers in canopied populations (max temperature: *r*^2^ = 0.24, *p* = 0.08, df = 1, 11) (Figure 6B) (min temperature: *r*^2^ = 0.20, *p* = 0.11, df = 1, 11) but no correlation was found for those populations in the exposed habitats (mean-max temperature: *r*^2^ = 0.04, *p* = 0.47, df = 1, 11) (mean-min temperature: *r*^2^ = 0, *p* = 0.79, df = 1, 11).

Minimum RH was a critical determining factor on larval numbers (*r*^2^ = 0.35, *p* = 0.03, df = 1, 11) (Figure 6C). Within the individual cohorts, mean-min RH during the larval phase had a marginally significant impact on the overall larval numbers (*r*^2^ = 0.23, *p* = 0.09, df = 1, 11) (Figure 6D). Mean-min RH also had a much greater influence on larvae in exposed habitats (*r*^2^ = 0.28, *p* = 0.05, df = 1, 11) (Figure 6E) than on canopied habitats (*r*^2^ = 0.06, *p* = 0.41, df = 1, 11). The correlation between precipitation and larval population numbers was observed only within the canopied habitat. The slope in this case was negative (*r*^2^ = 0.21, *p* = 0.11, df = 1, 11) (Figure 6F).

## 4. Discussion

Many previous ecological studies were conducted on fever tick populations in tropical Queensland. In this field study, over two years in south Texas, we documented recurring seasonal population patterns that were habitat-dependent. We confirmed that mean-min RH (saturation deficit) had the greatest influence on larval survivability in the exposed habitats. Previous laboratory studies showed that minimum RH is the determining factor of larval survival, regardless of temperature [35], with reports of minimum RH (≤63%) decreasing larval survival time by 53–72 days [35]. It was observed that *R. australis* larvae survived less than 15 days in RH levels below 65% [12]. It was also reported that *R. australis* larvae required high RH levels (≥95%) to replenish lost body moisture [36,37]. Notably, maximum larval longevity reached 115 days, which was attributed to the higher relative humidity in the shade [38].

In our study, as in previous studies, humidity is a key factor in survival, but our results show that minimum RH had a greater impact on larval survival in exposed habitats compared to canopied habitats. Interestingly, simulation models [8] predict a greater overall larval survival under canopied conditions regardless of seasonality. In the current study, we challenge this prediction based on our field results. In the summer season, we showed that the canopied larval population was significantly higher compared to exposed conditions as predicted by the models. However, contrary to simulated predictions we report that winter and spring had the highest larval populations in exposed conditions not canopied conditions. Conversely, *R. australis* larvae (in Queensland) had higher survival and increased longevity in the shade compared to exposed conditions in summer and winter [35]. Predictions from the simulation model [8] for *R. microplus* in canopied habitats agree with field data from Australia. As an explanation for this reversal, we hypothesize that the egg incubation phase takes longer in the higher latitude Texas winter, especially in the shade compared to exposed conditions. Warmed by the sun, development would be faster, and thus less time exposed to desiccation and predation. In tropical north Australian climates [39], Wilkinson and Wilson [35] reported that *R. australis* eggs still proceeded with development during their winter temperatures (11–21 °C) and humid conditions (69–70%) [36], meaning comparatively less time exposed to the environment. A shorter development time in the larval stage is advantageous because, unlike the vulnerable egg stage, the motile larvae are able to move to suitable microclimate habitats.

As one would expect, precipitation has a measurable effect on larval populations, but it was dependent on timing. Precipitation during the larval stage demonstrated no significant relationship to larval populations, but, there was a correlation with the incubation phase. Precipitation negatively correlated with reproductive success as indicated by the number of positive tick gardens. This might suggest that the amount of rain affected the egg stage of the cohorts. Although drowning of the eggs is a possibility [40], an increase in mortality from fungal pathogens is also possible [41]. Another possibility is breakup of the egg mass by heavy rains might have led ultimately to desiccation. For example, eggs of *R. australis* did not hatch with constant RH less than 70% [12]. It was reported that the mortality of eggs due to desiccation is a major determinant of larval tick number in *Rhipicephalus appendiculatus* [42] and *R. australis* [43].

In the comparison of habitats, ambient temperatures only had influence on larval survivability under the canopied habitats. This was positively correlated with tick numbers suggesting that RH in canopied habitats was within tolerance therefore allowing for temperature to have a detectable influence in canopied habitats. Ambient temperatures had no measurable effect on reproductive success under canopied habitats. Larvae of *R. microplus* could survive in lower temperatures if there are high RH levels [44]. With temperatures ranging from 15–29 °C, *R. microplus* and *R. australis* larvae have been recorded to have prolonged survival time [12,36,37]. This range might establish a threshold that influences population survival under the canopied habitats during the winter and spring. In contrast, larval populations decreased if directly exposed to high temperatures with high RH [36,37]. With high midday temperatures, 40% of *R. microplus* larvae would descend to soil levels then reascend to quest by early evening [45]. During the seasons with the highest temperatures, plants that provide good cover are crucial for the survival of the larvae [1,46]. In this experiment, the exposed habitats contained the lowest population sizes during the summer and fall. We agree that, with the increase in the density of vegetation, the atmospheric conditions become less important [47].

Overall, the fall cohorts exhibited the lowest population size compared to the other three seasons. With regards to low population sizes, possible contributing factors are as follows: percentage of female oviposition comparatively low (54%), high ambient temperatures, low humidity levels, and low precipitation. It has been proposed that decreased daylight and low temperatures may possibly inhibit questing activity [16]. In our study, spring had the highest female oviposition success (88%) and subsequently the highest number of larvae. This peak has been labeled the “spring rise” [48]. This phenology is not shared by all ticks. In contrast to this phenomenon, *Amblyomma cajennense* larvae were not observed to be questing in the spring (November) or summer (January) in Brazil [49]. However, larvae of *Ixodes scapularis* were recorded to remain actively questing from April to October [50]. Larvae of *Ixodes dammini* were most abundant in the late summer and early fall [51].

## 5. Conclusions

Our study supports previous predictive and field studies identifying the relationship that seasonality and habitat have with larval fever tick survival. We showed that *R. microplus* survival in south Texas varied among seasons and was dependent on the habitat. For closing the information gap on *R. microplus* larvae, better off-host control can be implemented. This study can provide program managers and the scientific community with knowledge about how larval population dynamics respond to the interaction between seasonality and habitat. For example, it will inform researchers where the larvae are most abundant—in either canopied or exposed habitats depending on the season. In addition, it will provide information on which abiotic factors have maximum and minimal influence on the larvae, in regards to seasonality. This study also provides results based on natural conditions reinforcing model predictions and laboratory studies done previously on *R. microplus*. This information, in return, can provide the foundation for future ecological studies on *R. microplus* larvae.

## Figures and Tables

**Table 1:** Larval tick numbers in relation to season and habitat. Collective (over two years) results comparing the mean ± standard deviation ($\bar{X}$ ± SD) seasonal numbers by habitat.

Habitat	Fall $\bar{x}$ ± SD	Winter $\bar{x}$ ± SD	Spring $\bar{x}$ ± SD	Summer $\bar{x}$ ± SD
Canopied	7.3 ± 15.6 ^A^	27.5 ± 71.8 ^A^	76.7 ± 179.4 ^B^	111.6 ± 193.1 ^B^
Exposed	5.4 ± 9.9 ^A^	162.3 ± 250.5 ^B^	263.2 ± 540.8 ^B^	10.3 ± 19.5 ^A^
All gardens	6.6 ± 19.5 ^A^	72.9 ± 166.6 ^B^	130.5 ± 3321^B^	81.2 ± 167.9 ^B^
Positive gardens only	11.8 ± 22 ^A^	93.1 ± 183.6 ^B^	147.5 ± 349.9 ^B^	115.94 ± 191 ^B^

Statistical comparison of means was by pair-wise *t*-test. Means followed by the same letter are not significantly different at *p* = 0.05. Means followed by the letter A and the letter B are significantly different from one another.

**Table 2:** Larval tick samples by individual cohort. For each cohort: season, number of gardens, number and percent of positive gardens, total larvae, mean number of larvae, and mean number of larvae in canopied and exposed habitats, respectively.

Cohorts	Season	N Gardens	Positive Gardens		Total Larvae	($\bar{x}$) Larvae	($\bar{x}$) Larvae Canopied	($\bar{x}$) Larvae Exposed
			N	(%)				
1	Winter	10	9	90	2185	218.5	13.8	123.2
2	Spring	16	15	93	4801	300.6	169.9	586.4
3	Summer	16	4	25	209	13.1	13.5	12
4	Summer	16	14	87	1188	74.3	84.9	6.4
5	Fall	16	12	75	107	6.7	7.3	5.4
6	Fall	16	0	0	0	0	0	0
7	Winter	16	8	50	385	24.1	2.4	71.8
8	Winter	16	16	100	1016	63.5	45.5	78.3
9	Spring	18	15	83	1598	88.8	54.7	319.6
10	Spring	18	16	88	387	21.5	19.8	77.4
11	Summer	18	18	100	2662	147.9	200.1	12.2
12	Fall	18	7	38	107	5.9	8.2	21.4
13	Winter	18	14	77	789	43.8	52.6	157.8
